# Supplementary material for: PCYT1A Missense Variant in Vizslas with Disproportionate Dwarfism
Source: Genes (Basel). 2022 Dec 13;13(12):2354. doi: 10.3390/genes13122354 (PMC9777673; doi:10.3390/genes13122354)
Supplement: Supplementary file 1 [file genes-13-02354-s001.zip › Figure_S1_flowchart_R1.pdf]

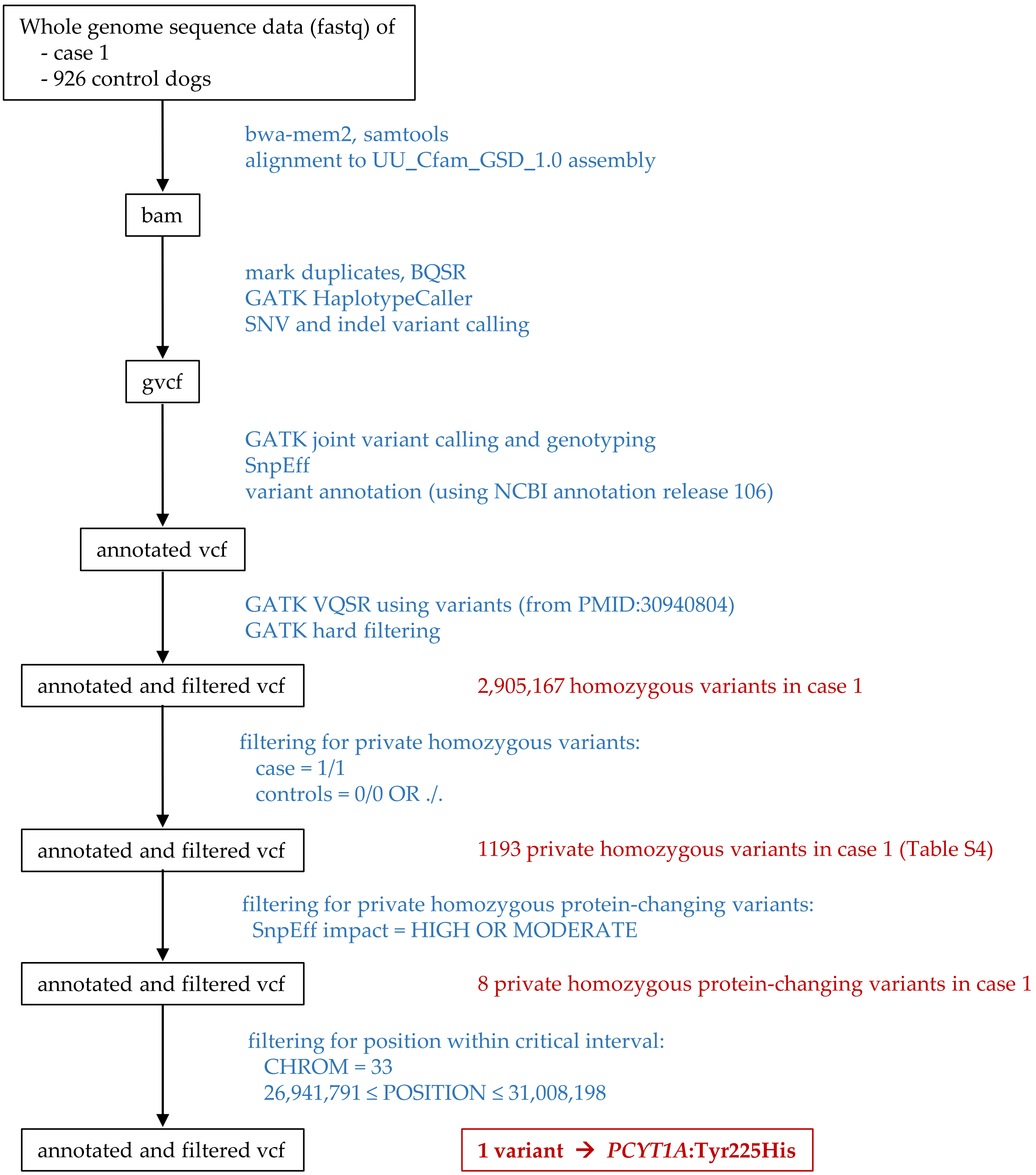

Figure S1. Flowchart illustrating the bioinformatic analysis steps for the identification of the candidate causative variant for skeletal dysplasia 3 in a Vizsla with disproportionate dwarfism. Software and analysis steps are indicated in blue, results are indicated in red. The complete pipeline with parameters can be found here: https://github.com/jmkidd/dogmap
