# Supplementary material for: PCYT1A Missense Variant in Vizslas with Disproportionate Dwarfism
Source: Genes (Basel). 2022 Dec 13;13(12):2354. doi: 10.3390/genes13122354 (PMC9777673; doi:10.3390/genes13122354)
Supplement: Supplementary file 1 [file genes-13-02354-s001.zip › Figure_S2_Clinical_photos_case8_R1.pdf]

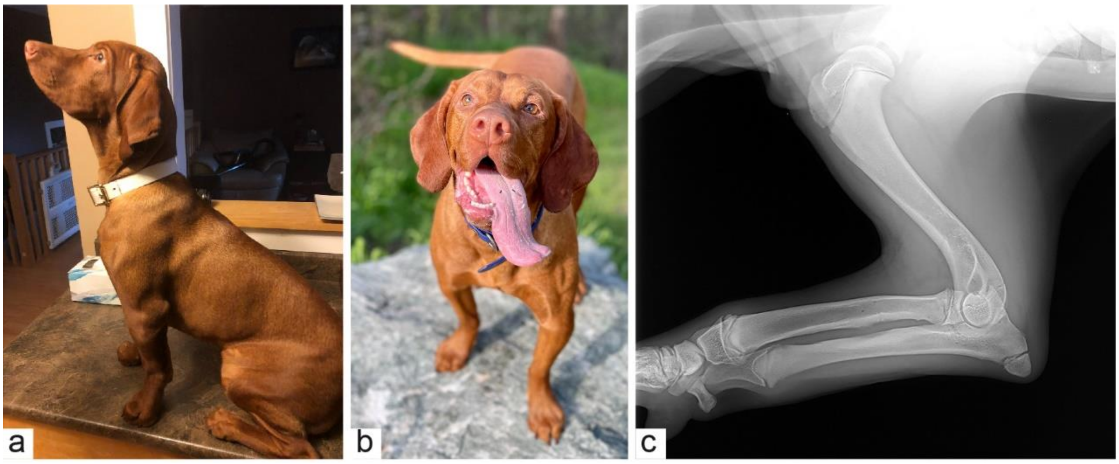

Figure S2. Phenotype of case 8. Clinical pictures and radiographs of a Vizsla with very short legs and significantly shortened lower jaw that was originally considered to be affected with the same form of disproportionate dwarfism as the other seven cases in our study. However, it turned out that this dog was homozygous wildtype (T/T) at the associated *PCYT1A*:c.673T>C variant. Thus, we assume that the phenotype in this dog, including the jaw malformation, is caused by different genetic and/or environmental factors (heterogeneity and/or phenocopy). **(a)** Short mandible. **(b)** Angular limb deformity. **(c)** Mediolateral radiograph of the right forelimb showing a short and thickened humerus, radius and fibula.
